# Modelling the correlation between the activities of adjacent genes in drosophila

**DOI:** 10.1186/1471-2105-6-10

**Published:** 2005-01-19

**Authors:** Helene H Thygesen, Aeilko H Zwinderman

**Affiliations:** 1 Clinical Epidemiology and Biostatistics, Academisch Medisch Centrum, University of Amsterdam, Meibergdreef 9, 1100 DD Amsterdam, Netherlands

## Abstract

**Background:**

Correlation between the expression levels of genes which are located close to each other on the genome has been found in various organisms, including yeast, drosophila and humans. Since such a correlation could be explained by several biochemical, evolutionary, genetic and technological factors, there is a need for statistical models that correspond to specific biological models for the correlation structure.

**Results:**

We modelled the pairwise correlation between the expressions of the genes in a Drosophila microarray experiment as a normal mixture under Fisher's z-transform, and fitted the model to the correlations of expressions of adjacent as well as non-adjacent genes. We also analyzed simulated data for comparison. The model provided a good fit to the data. Further, correlation between the activities of two genes could, in most cases, be attributed to either of two factors: the two genes both being active in the same age group (adult or embryo), or the two genes being in proximity of each other on the chromosome. The interaction between these two factors was weak.

**Conclusions:**

Correlation between the activities of adjacent genes is higher than between non-adjacent genes. In the data we analyzed, this appeared, for the most part, to be a constant effect that applied to all pairs of adjacent genes.

## Background

Several studies (Hamilton [1], Fukuoka[2]) have found stronger correlation between the expression levels of genes that are located close to each other on the genome than between those of distant genes: when gene expressions of many genes are measured for multiple tissue samples, for example using microarray technology, adjacent genes are sometimes found to be consistently up- or downregulated in a subset of the tissue samples.

Gene expression is influenced by many factors (for a review, see Orphanides[3]), many of which could influence the correlation between the expression of two genes in general, and that between two adjacent genes in particular. Of particular interest are *chromatin domains*. DNA can exist in either one of two states: a condensed state, termed heterochromatin, which is broadly inaccessible to transcription (although there are exceptions (Orphanides[3])), and an active state, termed euchromatin. A *chromatin domain *(a segment of DNA which, in a given cell at a given moment, is either entirely euchromatin or entirely heterochromatin) typically spans several genes (Roy[4]). Therefore, one would expect the expressions of two adjacent genes to tend to be positively correlated, at least if it was possible to measure transcription in individual cells. If the chromatin state was completely random (Jackson[5]) suggested a dynamic equilibrium, where chromatin fluctuates, to some extent randomly, between the two states), the effect of chromatin domains would vanish when gene expression is measured in pools of many cells, as with microarray technology. However, there is ample evidence for non-randomness. For example, chromatin states tend to be preserved after cell division (Orphanides[3]). And Cho[6] demonstrated that the states of chromatin domains in yeast are related to the cell cycle.

In addition to the chromatin theory, several other explanations have been suggested for the apparent correlation between the expressions of adjacent genes. Several authors (Cohen[7], Kruglyak[8]) have noted that divergent gene pairs show stronger correlation than tandem and convergent pairs, possibly because divergent pairs share an Upstream Activation Sequence. Lercher[9] found that many of the co-expressed adjacent genes in *Caenorhabditis elegans *are either operons or homologues (see also Llorente[10] and Rossfll]), and it has been suggested that evolution has arranged for functionally related genes to be located close to each other, either in order to promote consistent inheritance (Bleiweiss[12]), or in order to benefit from the correlation accounted for by the chromatin domains (Cohen[7]). Parisi[13] found a nonrandom distribution of the chromosomal location of genes with high expression level in testis and ovaria in Drosophila. Jackson[5] suggested that the location of a gene in the nucleus plays a role for its transcription, in relation to gradients of the concentration of transcription factors.

Finally, since the action of a transcription factor on a promotor gets weaker with distance, genes belonging to the same pathway should show stronger correlation if they are located close to each other (Dorsett [14]). Due to this abundance of alternative theories, a study of gene-expression correlations should be designed in a way that makes it possible to distinguish correlation structures predicted by one model from those predicted by other models. The same applies to the statistical analysis techniques used.

An important consequence of the evolution-based theories is that they predict a *consistent *coregulation structure. Suppose that two genes (in this case, two adjacent genes) are co-regulated because, for example, they participate in the same pathway. They would, then, show a strong correlation because they would be co-regulated in all tissue samples. This need not necessarily be the case with the chromatin domain model: the segments of euchromatin in one tissue sample may overlap with those in another tissue sample. This latter scenario, we call an *inconsistent *coregulation structure. With consistent coregulation, adjacent gene pairs will show either strong positive correlation, or they will be uncorrelated. With inconsistent coregulation, all adjacent gene pairs will show a modest positive correlation.

In a microarray analysis of gene expressions in 35 pools of drosophila embryos and 54 adult drosophilae (Spellman and Rubin[15], reviewed by Oliver[16]), it was shown that adjacent genes with correlated expression levels tend to cluster. The method they used to demonstrate this was the following: let *w *be a fixed window size, e.g. 10. For each window of *w *adjacent genes, the average pairwise Pearson correlation coefficient within the window was computed. If that measure was found to be significant at, say, 1 - *α *= 0.999 (the p-value was estimated in a permutation experiment), all the genes in the sequence were tagged. Doing this for all windows (they were allowed to overlap), the total number of tagged genes was counted. Then the experiment was repeated with shuffled genes (i.e., as it would behave in the absence of positionally related correlation), and the number of tagged genes in the shuffled experiment was subtracted from the number of tagged genes in the original experiment. This difference (called "net genes") grows with window size and starts plateauing for a window size of approximately 10. Spellman and Rubin interpreted this as evidence for gene interaction within regions of approximately that size.

**Figure 1:**
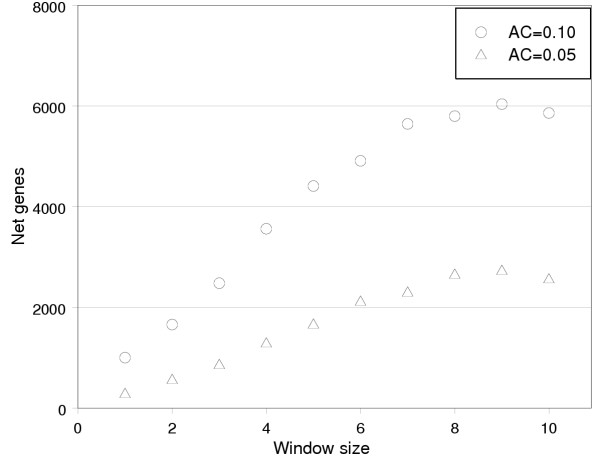
**Net genes for simulated data. **Number of genes that contribute to a high moving average of Pearson R in simulated data, as a function of the size of the windows used for computing the moving average. The shapes of the curves are similar to the findings by Spellman and Rubin, although the convergence is somewhat faster. The simulated data are first-order Gaussian autocorrelation processes.

One problem with the above method of analysis is that the increasing number of "net genes" would occur even without direct interactions between genes separated by up to ten positions. As shown in figure 1, the analysis gives similar results when applied to simulated data from a normal distribution, in which an autocorrelation of AC = 0.10 or 0.05 was imposed artificially. So we cannot, on the basis of the analysis described above, reject the hypothesis that the data arose from a simple first-order autocorrelation process, in which no clustering of correlated genes exists. It is true that gene-pairs with high correlation form clusters: the autocorrelation of Pearson's R for adjacent genes is 0.1, with a standard error 0.01. However, this can be explained by the fact that genes that tend to correlate strongly with other genes in general (for example because of low measurement noise) tend to correlate with both their neighbors. If one eliminates that confounder by looking at non-overlapping gene pairs only, the autocorrelation vanishes (0.01, standard error = 0.01). Another way of showing this is by means of cross-tables. We divided the adjacent gene-pairs into three groups: positively correlated pairs(R>0.7), negatively correlated (R< -0.7) and non-correlated. (The threshold of 0.7 was suggested by Cohen[7]). If the correlated gene-pairs were clustered, one would expect that a gene-pair belonged to the same group as the next gene-pair more often than would happen by chance. This is indeed the case when overlapping gene-pairs are considered: 627 gene pairs out of 12949 (4.8%) had an R > 0.7 while the next (overlapping) gene pair also had an R > 0.7. This is 2.22 times more than what we expected due to chance alone. However, the same was observed when only one of the two overlapping gene-pairs was was a neighbor pair and the other was a random pair (if the genes were labelled ABCD...Z, a strong correlation between A and B predicted a strong correlation between B and C but also between B and X, where X is a random gene). But when non-overlapping adjacent gene pairs were considered (say, AB versus CD), the contingency was 332 out of 12878(2.6%) which is only 1.18 times more than expected due to chance. So the apparent clustering of correlated gene pairs is mainly due to overlap rather than to adjacency.

On the other hand, it is clear that there is some higher order correlation structure in Spellman and Rubin's data. This can be seen by computing the average correlation coefficient for subgroups of the gene pairs, based on their physical distance (table 1) – it decreases much slower with distance than would a first-order process. Hence, the question remains how the correlation structure should be modelled and analyzed. In this paper, we present a method to separate

A) Correlation of gene expression that can be attributed to consistent coregulation, from

B) The uniform correlation expected under an hypothesis of inconsistent corregulation.

## Results

### Data

We used the Drosophila data published by Spellman and Rubin[15]. This data set consisted of normalized expression levels of 13090 genes in 89 flies (35 pools of embryos and 54 pools of adults), obtained with Affymetrix GeneChip microarrays. For the purpose of analysis based on physical distance between genes, the data set was linked to Flybase[17] on the basis of the CG-identifiers provided by Spellman and Rubin. 1871 genes had to be omitted from that analysis because of unmatched CG-identifiers. We checked that these omitted genes were not a biased sample with respect to correlation in expression with their neighbor genes.

### Non-adjacent gene pairs

The distribution of the correlation coefficient between gene pairs in general, (i.e., genes that are not necessarily adjacent) was fitted with a three-component normal mixture, based on the Arcus Tangens Hyperbolic-transform (Fisher's z) of the correlation coefficients, which was validated with a Kolmogorov-Smirnov test (p = 0.6). As shown in table 2, the mean of the middle component, tanh(*μ*_0_), was 0.014 with a standard error of 0.002. (In all tables, *μ *has been transformed back with tanh so that it can be interpreted as a correlation). The fact that the standard deviation of the third component, *σ*_+_, is greater than that of the other components, suggests that the co-regulation of some gene pairs was stronger than that of others.

### Adjacent gene pairs

**Figure 2:**
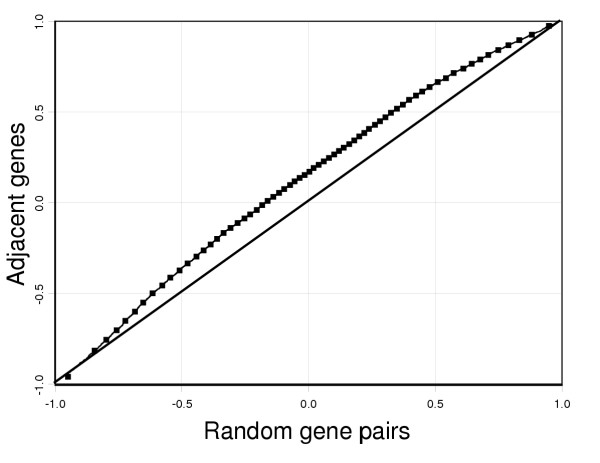
**Pearson R for adjacent genes in Spellman and Rubin's data set, compared to non-adjacent genes. **The QQ-plot is shifted away from the diagonal over the whole correlation spectrum, suggesting that co-regulation applies to all pairs of adjacent genes.

**Figure 3:**
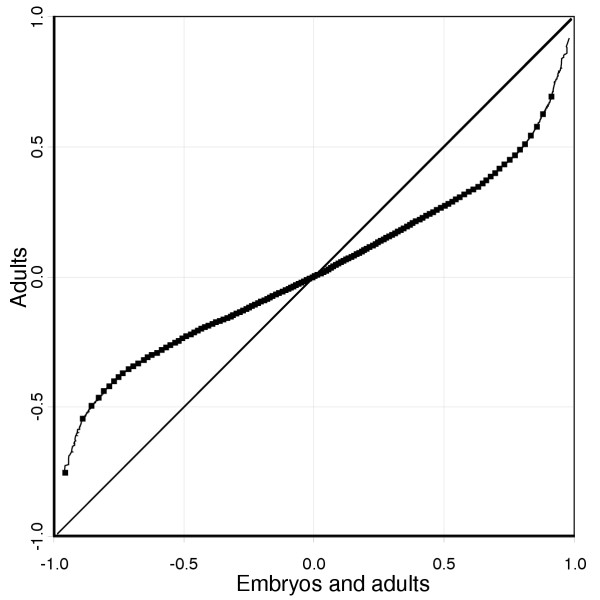
**Pearson R for non-adjacent genes in the adult flies, compared to a mixed fly group of the same size (54 flies). **The average correlation is the same in both fly groups, but strong positive and negative correlation occurs in the mixed group. This is consistent with the assumption that strong correlation of two genes can occur when both genes are upregulated (or downregulated) in one age group relative to the other age group.

Table 3 shows the fitted parameters in the same model, but for adjacent gene pairs. The results are very similar to those for the random gene pairs, although *μ*_0 _and *μ*_+ _are substantially higher. This suggests that the effect of adjacency is, for the most part, a mechanism that applies to adjacent-gene pairs in general, not just to specific adjacent-gene pairs. One way to illustrate this is by means of a qq-plot (figure 2), which shows that the correlation for random gene pairs and adjacent gene pairs have a similar structure, although the overall correlation is stronger in adjacent gene pairs. This contrasts with the qq-plot in figure 3, in which the gene-expression correlations in the subset of adult flies is compared to those in a mixed fly group. On average, the correlation is zero in both the adult group and the mixed group. However, the standard deviation of the correlation is much higher in the mixed group, presumably because genes that are expressed specifically in the adults (or specifically in the embryos) correlate strongly with each other in the mixed group.

Table 4 shows the fitted values of the parameters *μ*_0_, *μ*_+ _and the size of the third component (that is, the fraction of the genes that are positively co-regulated), for random and adjacent gene pairs in four subsets of the flies: one containing all 54 adult pools, one containing all 35 embryo pools, and two random subsets of 35 and 54 pools, respectively. One can observe that *μ*_0 _(which can be attributed to corregulation of gene pairs in general, i.e., inconsistent co-regulation) is always high for adjacent gene pairs and low for random gene pairs. On the other hand *μ*_+ _and the "+"-fraction (which can be attributed to corregulation of specific gene pairs, i.e., consistent co-regulation) are always high in heterogenous fly groups and low in homogenous one. That difference between heterogenous and homogenous groups is what we expected: In a homogenous fly group, less gene pairs will come up as co-regulated, because some pathways may either be active in all flies, or inactive in all flies. That age groups does not explain all correlation, is hardly surprising: although the adult group and the embryo group are less heterogenous than the mixed group, neither is homogenous.

### Gene pairs of intermediate distance

To get an impression of the size of the segments involved in co-regulation, we fitted the three-component mixture to the correlation coefficient between the expressions of more distant gene pairs. *μ*_0 _was 0.114 for the correlation between the expression of a gene and that of its direct neighbor, and 0.08 for the correlation with its second neighbor. From the fifth neighbor and until at least the tenth, a stable level of 0.03 is reached, which is still higher than for distant gene pairs. One possible interpretation of this is that two different co-regulation effects exist: a short-segment effect accounting for a correlation of approximately 0.114-0.03 = 0.081, and a long-segment effect accounting for a correlation of approximately 0.03-0.014 = 0.016. At first we had the suspicion that this stable level of 0.03 was a whole-chromosome effect, but that was not the case. We found no significant difference between the overall average correlation of random gene pairs from the same chromosome and random gene pairs from different chromosomes.

In a first-order autocorrelation process, tanh(*μ*_0_) for the second neighbor would be the square of that for the first neighbor. However, the unexpectedly small difference between the two values could be due to the fact that some second neighbors are physically quite close. To confirm or reject the hypothesis of a first-order process, however, the analysis must be based on physical distance rather than simple adjacency. As shown in table 1, the decrease in average correlation as a function of physical distance is still too slow for a first-order process.

Table 5 shows the fitted parameters for gene pairs within a physical distance of between 100 and 1000 bases. Unlike the fitted parameters for neighbor genes, *μ*_+ _is not significantly higher than for random gene pairs.

### Simulated data

To validate our approach, we computed the correlation coefficients of adjacent and random gene pairs in simulated data, based on either consistent or inconsistent co-regulation. As expected, the simulated data with inconsistent co-regulation resulted in a correlation structure with only one component, whereas those with consistent coregulation showed two components. (Not three, since the *segment effect *(see Methods) was always positive in the simulated models).

## Discussion

There are many theories that could explain correlation between the expressions of two genes in general, and that of two adjacent genes in particular. For the verification of the specific correlation structures predicted by each theory, Spelmann and Rubin's data turned out to be very useful. Their experimental design was based on two distinct classes of experimental conditions (in this case, embryos and adults), which makes a relatively simple correlation structure plausible. Also, the data from each of their microarrays could be fit nicely with a normal mixture, which makes it possible to analyze their data on the basis of the Pearson R. As expected, most of the consistent correlation was found to be related to age group and unrelated to adjacency. Maybe more surprisingly, most of the correlation that was related to adjacency could not be accounted for by consistent co-regulation. This suggest that, at the statistical level, co-regulation of adjacent genes should be understood in terms of the mechanics of the transcription process, rather than in terms of the evolutionary origin of specific gene groups. In other words, in this particular data set, consistent correlation was very widespread, applying to 40% of all gene pairs (table 2), half of this could be attributed to the two age groups (table 3), but this consistent correlation was not the most important contributor to the elevated correlation coefficients for adjacent gene pairs. Correlation between the expression of adjacent genes is evident, but it is possible, as suggested by Spellman and Rubin, that such a correlation between expression levels for two adjacent genes does not generally imply a relationship with respect to biological function.

Spellman and Rubin concluded that the adjacent genes with correlated expressions were confined to certain domains, which spanned 20% of all genes. Our findings are different, in that we distinguished between two components of the correlation structure. We found a baseline correlation of 0.1 (the difference between *μ*_0 _in table 2 and table 3) applying to all adjacent gene pairs. In addition to that, we found that 8% of all adjacent gene pairs (the difference between the "+" -fractions in table 2 and table 3) were strongly positively correlated. Finally, unlike Spellman and Rubin, we found no evidence for clustering of the strongly correlated gene pairs.

Since this data set contained data from whole animals, we cannot say anything about the role of chromatin domains (or other mechanisms causing correlation of the expression of adjacent genes) in tissue differentiation. Parisi et al. [13] found clustering of genes that were upregulated in drosophila germ cells, which suggests that one would find elevated consistent coregulation of adjacent genes when applying our model to data sets with different tissue classes.

Even for random (non-adjacent) gene pairs, *μ*_0 _was slightly positive. It is hard to imagine a biological effect that could cause *μ*_0 _to be significantly greater than zero, but it could be an artifact of the normalization: if gene *g *is expressed at the same level in all tissue samples, and gene *h *as well, they should have a correlation coefficient of zero, but since they will both yield elevated measurements in flies in which the normalization bias is positive, the observed correlation will be positive. The fact that *μ*_0 _is almost zero suggests that normalization bias is not a major problem with this data set. The fact that *μ*_0 _for random gene pairs is not generally higher in mixed age groups than in pure adult or embryo groups (table 4) is consistent with the assumption that *μ*_0 _for random gene pairs does not have a biological interpretation. Although looking at adjacent genes is computationally convenient, it would probably be more correct to use physical distance, rather than adjacency, as a covariate. Actually, Fukuoka[2] found that physical distance is stronger related to correlation of expression levels than is adjacency. Table 5 shows that when a subgroup of the gene pairs is selected on the basis of physical distance, it becomes even more clear that the elevated correlation is a general property of all near-by gene pairs. This is not surprising since the group of adjacent gene pairs is heterogenous, containing gene pairs with vastly varying distances. Unfortunately, there are some problems related to the use of physical distance.

First, it is not clear with respect to which anchor the physical distance should be defined. We have chosen to use the minimal distance (using the end nearest to the neighbor as an anchor), because it is not confounded by the length of the gene. However, depending on which co-regulation mechanism one has in mind, other distance measures might be more natural. This question becomes crucial if one wants to make subgroup analysis based on convergent, divergent and tandem gene pairs.

Second, physical distance is a continuous variable, which means that the model must be augmented with an assumption regarding the relationship between distance and correlation.

The question remains to what biological phenomena the baseline correlation between adjacent genes should be attributed. Several of the theories that have been proposed could account for it, and it is likely that several mechanisms play a role.

In addition to the baseline correlation, we also found more consistent correlation between adjacent genes and inconsistent co-regulation, but this does not necessarily imply a combination of two mechanisms: one could imagine a kind of quasi-consistent mechanism, in which groups of adjacent, co-regulated genes could have boundaries everywhere, but with some boundary locations being more likely than others. Finally, it has been suggested that the correlation between the expressions of adjacent genes should be understood in terms of enhancer-promotor interaction, which weakens with distance(Dorsett[14]). It is possible that such a model would predict a pattern similar to what we have found.

## Conclusions

It is possible to analyze correlation structures in gene expression data on the basis of simple, parametric models with known mathematical properties and parameters with biological interpretations, or at least some candidate interpretations. When applied to the data from Spellman and Rubin, it appeared that the expressions of two genes can show positive correlation for either of two largely distinct reasons: because they share some confounder unrelated to adjacency (this is often the age group, but could also be some other biological parameter, or a technical artefact), or because they are located close to each other on the chromosome. The underlying biological mechanisms remain unknown, but there appears to be a component of the correlation that depends on distance only and not of the biological function of the genes. If this is true, gene clustering algorithms might benefit from making distinction between the adjacency-related and not-adjacency-related co-regulation, for example by subtracting the effect of adjacency from the correlation of adjacent genes. Doing that would, according to our findings, lead to more reliable identifications of gene pairs with related biological functions.

## Methods

### Non-adjacent gene pairs

First, we constructed a model for those co-regulation effects that were unrelated to the relative location of the two genes. Suppose that a gene can be active either in adults, in embryos, in both, or in neither. Let *Y*_*gi *_be the log-scale measured activity of gene *g *in fly *i*, fly *i *having development stage *s *(either "adult" or "embryo"). A natural model for *Y*_*gi *_would be a two-component normal mixture:


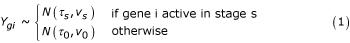


Since the data were normalized so that the average logscale activity across all 89 flies was zero, the activity of gene *g *in the other development stage should be taken into account. We therefore assumed a three-component normal mixture:


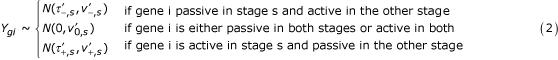


Actually, of the 89 flies, the gene expressions in 87 of them could be nicely fitted with a three-component, normal mixture, while in two of the adult flies we identified only two components (the component of genes that were passive in adults while active in embryos vanished in those two cases).

If the three-component model is correct, Fisher's z-transform, which is the hyperbolic arcus tangens of the Pearson correlation coefficient *R*_*gh *_for two genes, *g *and *h*, is


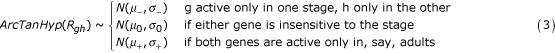


We expected *μ*_0 _to be close to zero. This model was validated by implementing it on the neighbor correlations in a *shuffled *data set, that is, for each gene its Pearson R with a random gene was computed. This procedure was repeated with 10 shuffled data sets, each generated by shuffling the genes in the original data set and subsequently computing the Pearson R for each pair of adjacent genes. For each shuffled data set, the parameters in the model were estimated with the EM algorithm (Dempster et.al[18]).

### Adjacent genes

Second, the distribution of the correlation coefficients of the pairs of adjacent genes in the unshuffied data set were compared to the same distribution for the shuffled data sets. Doing that, we could distinguish two different contributions to the correlation; A) co-regulation effects that are unrelated to the fact that two genes are adjacent, and B) co-regulation effects related to the fact that two genes are adjacent. We also analyzed each gene's correlation with its second, third etc. (up to the tenth) neighbor, in order to get an idea of the lengths of the chromatin domains in question.

Third, we applied the above two procedures to two subsets of the data, namely the data for adult flies and the data for embryos. If our assumption, that all (or at least most) correlation could be attributed to the development stage, one would expect much less correlation in those subsets. However, those subsets differ from the entire data set by the mere fact that they are smaller. Therefore, we also applied the same procedure to two random subsets of 35 and 54 flies, respectively, stratified by development stage.

### Physical distance

For those gene pairs where the start and the end of the open reading frame (ORF) was available, we defined the physical distance as the distance between the end of the ORF of the first gene and the start of the ORF of the second gene. We chose that definition because other distance definitions (defined on the basis of the direction of transcription, or on the basis of mid-points) would be confounded by the length of the ORFs.

### Simulated data

Finally, in order to validate our method, we applied it to 6 simulated data sets, which we simulated on the basis of the two alternative hypothesis of consistent and inconsistent co-regulation. The simulated data sets contained 100 tissue samples and 1000 genes. The genes were randomly divided into either 67, 200 or 500 segments, corresponding to average segment sizes of 15, 5 and 2. This means that each gene belonged to exactly one segment. For each average segment size, we simulated one data set with *consistent co-regulation*, in which the same segments were used for all 100 tissue samples, and one data set with *inconsistent co-regulation*, in which a separate segmentation was sampled for each tissue sample. If gene *g *belongs to segment *s *in sample *i*, the expression *Y*_*gi *_was given by

*Y*_*gi *_= *x*_*si *_+ *ε*_*gi *_    (4)

where *x*_*si *_+ and *ε*_*gi *_were both sampled from the standard normal distribution. Notice that this model does not account for different tissue classes, so it should be compared to the results from the adults-only and embryos-only subsets. The error term *ε*_*gi *_should be interpreted as a combination of measurement errors and biological effects that are unrelated to the segmentation.

## Authors' contributions

HT did the explorative data analysis and suggested the concept of consistent co-regulation and using three-components mixtures for the correlation structure. AZ suggested using Fisher's z-transform for the correlations. HT did the literature review. Both authors contributed to the manuscript.

## Figures and Tables

**Table 1:** Average correlation between gene pairs of different physical distances. The distances are minimal distances in bases.

Distance	Mean R	SE(R)
Overlapping	0.10	0.01
0–999	0.17	0.01
1000–1999	0.14	0.01
2000–2999	0.12	0.01
3000–3999	0.10	0.01
4000–4999	0.08	0.01

**Table 2:** Random gene pairs. Fitted parameters in the three-component mixture of ArcTanH(R) for random gene pairs. *μ *has been back-transformed so that it can be interpreted as a correlation.

Component	fraction	SE (fraction)	*μ*	SE(*μ*)	*σ*	SE(*σ*)
-	.1687	.0007	-.660	.001	.332	.002
0	.5955	.0006	.014	.002	.330	.001
+	.2352	.0008	.571	.002	.439	.002

**Table 3:** Adjacent genes. Fitted parameters in the three-component mixture of ArcTanH(R) for adjacent genes. *μ *has been back-transformed so that it can be interpreted as a correlation.

Component	fraction	*μ*	*σ*
-	.118	-.647	.312
0	.571	.114	.344
+	.311	.655	.480

**Table 4:** Subsets of the flies. Parameters in the three-component mixture of ArcTanH(R) for different subsets of the flies. *μ *has been back-transformed so that it can be interpreted as a correlation.

Flies	Gene pairs	*μ*_0_	*μ*_+_	"+" fraction
35 embryos	Adjacents	0.09	0.44	0.21
35 embryos	Random	0.03	0.30	0.16
35 random	Adjacents	0.11	0.68	0.32
35 random	Random	0.02	0.60	0.25
54 adults	Adjacents	0.08	0.37	0.18
54 adults	Random	0.01	0.25	0.15
54 random	Adjacents	0.12	0.65	0.31
54 random	Random	0.08	0.57	0.24

**Table 5:** Physically close gene pairs. Fitted parameters in the three-component mixture of ArcTanHyp(R) for gene pairs within a distance of between 100 and 1000 bases from each other. *μ *has been back-transformed so that it can be interpreted as a correlation.

Component	fraction	*μ*	*σ*
-	.11	-0.66	.34
0	.64	0.14	.46
+	.25	0.74	.49
